# On the Demographic and Selective Forces Shaping Patterns of Human Cytomegalovirus Variation within Hosts

**DOI:** 10.3390/pathogens7010016

**Published:** 2018-01-28

**Authors:** Andrew M. Sackman, Susanne P. Pfeifer, Timothy F. Kowalik, Jeffrey D. Jensen

**Affiliations:** 1 School of Life Sciences, Arizona State University, Tempe, AZ 85281, USA; sackman@asu.edu (A.M.S.); susanne.pfeifer@asu.edu (S.P.P.); 2 Department of Microbiology and Physiological Systems, University of Massachusetts Medical School, Worcester, MA 01655, USA; timothy.kowalik@umassmed.edu

**Keywords:** human cytomegalovirus, population genetics, viral evolution

## Abstract

Human cytomegalovirus (HCMV) is a member of the β-herpesvirus subfamily within Herpesviridae that is nearly ubiquitous in human populations, and infection generally results only in mild symptoms. However, symptoms can be severe in immunonaive individuals, and transplacental congenital infection of HCMV can result in serious neurological sequelae. Recent work has revealed much about the demographic and selective forces shaping the evolution of congenitally transmitted HCMV both on the level of hosts and within host compartments, providing insight into the dynamics of congenital infection, reinfection, and evolution of HCMV with important implications for the development of effective treatments and vaccines.

## 1. Introduction

Human cytomegalovirus (HCMV) is a β-herpesvirus in the Herpesviridae family of dsDNA viruses. It possesses the largest genome of any known human virus (~235,000 bp), and is nearly ubiquitous in human populations, with a seroprevalence of 30–90% in the United States and >90% in adults outside of the developed world [1,2]. Though HCMV infection is generally asymptomatic or mild in most individuals, it can lead to severe symptoms in immunocompromised patients and neonates [3,4]. HCMV is the most common cause of birth defects resulting from an infectious agent, affecting ~0.7% of life births in the U.S. (30,000 per year), with 20% of congenitally infected infants exhibiting permanent neurological sequelae, including deafness, blindness, and/or mental disability [4].

HCMV is exquisitely adapted to its human host, and cytomegaloviruses in general demonstrate a high level of host-specificity, with non-human CMV being absent even in populations with high levels of opportunity for zoonotic transmission [5]. The long-term evolution of HCMV has been well documented [6,7], but the short-term evolution of HCMV within hosts has only recently been elucidated, and much remains unknown. We review recent work that has been devoted to characterizing the heretofore unanticipated abundance of within-host variation and the complex demographic, genetic, and selective forces that shape the populations of HCMV within congenitally infected newborns. We argue that further research into the population-level processes of transplacental congenital infection and subsequent dispersal and compartmentalization is necessary for progress to be made toward successful treatments or preventative vaccines.

## 2. HCMV Diversity within Hosts

A large body of work has previously shown that HCMV exhibits high levels of genetic diversity within individuals [8,9,10,11,12], a potentially unexpected finding, given that HCMV encodes a DNA polymerase with proofreading activity [13]. Most research has historically focused on the sequencing of just a few loci, such as glycoproteins gB, gN, gH, and g0 [14,15,16,17,18,19,20], and has generally suffered from technological limitations related to genome coverage and sequencing depth. However, several recent studies have applied modern high-throughput sequencing methods to analyze whole genomes of longitudinally time-sampled populations [21,22,23,24,25,26,27,28], yielding novel insights into the full extent of HCMV diversity by measuring the frequencies of even rare variants within populations of HCMV and elucidating patterns of population-level evolution following initial infection.

Renzette et al. performed population genomic sequencing from urine samples collected within two weeks of birth from three congenitally HCMV-infect neonates and found that intrahost pairwise diversity across all known open reading frames (ORFs) averaged 0.22%, similar to levels observed in genome-wide analyses of human immunodeficiency virus (HIV) and ORF-specific analyses of other RNA viruses, such as dengue and West Nile [21,29]. At the time, however, it remained unclear how this high level of diversity arose in light of the low mutation rates of herpesviruses relative to those of RNA viruses. 

Further work by Renzette et al. used similar population genomic analysis of urine and plasma samples from five congenitally infected infants to investigate divergence between individuals as well as within individuals across sequentially sampled time points and between different tissue compartments [22]. HCMV is known to disseminate across many tissues and organs of the body, and there is some evidence of variable levels of diversity within compartments [22,30] and genetic differentiation between specific tissues, possibly owing to cellular tropism [9,31,32]. Remarkably, they found that *F_ST_*, a measure of differentiation between populations, was roughly the same between populations from the urine and plasma compartments of the same patient as between populations from the urine compartments of two unrelated individuals, suggesting evolutionary forces of sufficient strength to generate rapid divergence between compartments within a single host [22]. Comparatively, within-host divergence of a single compartment appeared largely stable over time. Hage et al. likewise found, from analysis of longitudinal population genomic samples, that a majority of 20 transplant recipients and congenitally-infected neonates included in the study displayed stability within the plasma compartment, but that nearly every individual exhibited evidence of mixed infection [24]. Additionally, one patient from this study suffering from full blown HCMV retinitis exhibited major differentiation between samples from the plasma compartment and the vitreous humour, providing additional evidence of the potential for extreme compartmentalization with HCMV. 

Having established that there are high levels of within-host genomic diversity in HCMV across compartments, we must next seek to understand what combination of neutral and selective processes are generating these patterns, given that the observed levels of genetic diversity are on par with those of high-mutation rate RNA viruses.

## 3. Demographic Process Underlying Congenital Infection

**Figure 1:**
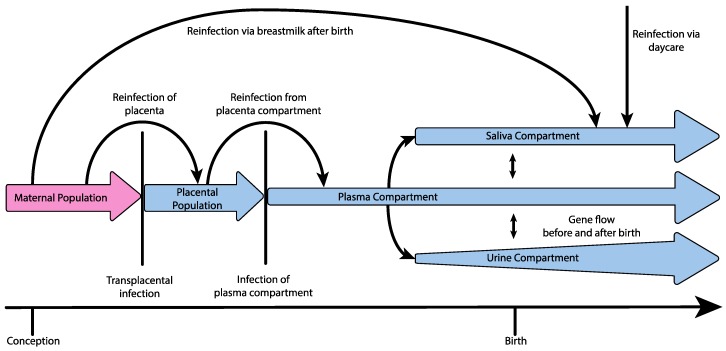
Hypothetical demography of congenital HCMV infection, including bottlenecks during infection of the placenta, infection of the plasma compartment from the placenta, and during the subsequent infection of additional compartments from the plasma (bottlenecks indicated by vertical lines). Evidence additionally suggests reinfection or continuous gene flow to the placenta from the mother over long periods of time, as well as reinfection of the plasma compartment from a stable placental population, gene flow between compartments within the new host before and after birth, rapid population growth within the urine compartment, and reinfection of the neonate via daycare or breastmilk.

One principle explanation for the high levels of observed diversity may be the demographic processes themselves underlying infection and dissemination within the host (i.e., the viral population history of size change, structure, and migration). Applying demographic modeling methods to time-sampled genomic HCMV data from five infants, Renzette et al. found support for a demographic model of transplacental infection and dissemination, wherein HCMV populations in congenitally infected neonates experience at least two bottlenecks *en route* to the kidney compartment, followed by subsequent population expansions within each compartment (illustrated in Figure 1, and see Table 1 for definitions of population genetic terminology). Specifically, at least one population bottleneck was inferred to occur during movement of the virus from the maternal compartment to the plasma compartment. Additionally, in one pair of twins, two bottlenecks were inferred during this step, including a bottleneck between 13–18 weeks of gestational age, possibly during transmission from the mother to the placenta, and a second bottleneck two weeks later during infection of the fetal plasma compartment, although it could not be determined whether the bottleneck during placental infection was an anomaly of that particular pregnancy [33,34,35]. After successful colonization of the plasma compartment, an additional bottleneck was inferred to occur during transmission from the plasma compartment to the urine compartment [22]. Perhaps most importantly, demographic models indicated that the bottleneck during maternal transmission across the placenta may involve as many as hundreds of unique HCMV genomes, generating a much greater initial pool of diversity than observed during RNA virus infection, which is generally thought to involve single or few virions [22,25,36,37]. Thus, the population history of HCMV infection may partly explain why measures of diversity in HCMV populations are comparable to those of RNA viruses, despite the lower mutation rate, although some evidence suggests that oral HCMV infection of infants may primarily occur via a single virus [38]. 

Multiple studies have also found support for the presence of gene flow between compartments after initial dissemination and colonization throughout the host as well as recurrent maternal admixture or reinfection, possibly seeding new diversity into individual compartments [22,28]. Pokalyuk et al. found, with longitudinal sampling of the plasma, urine, and saliva compartments, that admixture from maternal HCMV populations can take place after initial infection and compartmentalization either through additional transplacental infection or reinfection of the fetus over a period of several weeks or months from a population of HCMV multiplying within the placenta, and reinfection is also known to occur after birth by way of the mother’s milk [28,39,40]. Furthermore, Renzette et al. found support for a demographic model including gene flow between compartments from longitudinal sampling of one patient’s urine and plasma, suggesting that, following collapse of the plasma compartment population due to the effect of the host’s adaptive immune response or antiviral therapy, the plasma compartment could be reinfected by genetically differentiated HCMV from other compartments [22]. If this mechanism of gene flow or periodic reinfection from various compartments is common, effective treatment design will need to account not only for epitope diversity within the targeted compartments, but for diversity across the other host compartments as well.

## 4. The Role of Variable Mutation, Recombination, and Selection in HCMV Evolution

In addition, an important factor contributing to variation in genome-wide diversity observed in HCMV appears to be attributable to genome-wide variation in mutation and recombination rates. Relatedly, there is accumulating evidence for variable levels of linked selection as well—namely, background selection effects modifying levels of variation owing to the recurrent removal of linked deleterious variation, and genetic hitchhiking effects modifying variation owing to linkage with positively selected alleles [41,42,43]. Renzette et al. calculated genome-wide rates of genetic diversity, as well as rates of mutation and recombination in 500 bp windows across the genome for 48 longitudinal HCMV-positive samples from 18 patients, estimating a genome-wide average mutation rate of 2.0 × 10^−7^ new mutations per base pair per generation, ten-fold higher than estimates for herpes simplex virus type 1, but comparable to estimates for murine cytomegalovirus [23,44,45]. Variable single nucleotide polymorphism (SNP) diversity was found to correlate across the genome with both mutation rate and recombination rate. SNP diversity was also strongly correlated with protein function, with genes encoding DNA-processing enzymes, capsid, and tegument proteins being highly conserved and genes encoding envelope and glycoproteins being on average more diverse. They also found that samples from the plasma compartment were more highly constrained than those in the saliva or urine compartments and were enriched for SNPs within glycoproteins and regulatory proteins, a possible response to cellular tropism or host immune response [23]. Related studies also found evidence for positive selection acting on glycoprotein mutations, as well as a high degree of longitudinal variability in glycoprotein SNPs [22,24]. This result is consistent with the biological understanding of glycoproteins in contributing to pathogenesis through differential properties conferred by polymorphisms, which are known to facilitate immune evasion via antigenic variation and which may also play important roles in tissue tropism and protection from neutralizing antibodies [46,47]. 

Given that the genomic impacts of selection are largely determined by local recombination rates [41,42], recent work has found support for background selection being the dominant mode of linked selection governing SNP diversity in HCMV, particularly in regions of low recombination rate. Conversely, genetic hitchhiking owing to positive selection appears dominant at only a handful of loci, including those associated with virulence and the transition from acute to persistent infection [26,48,49]. In total, 23–28% of the HCMV genome appears to be strongly conserved as a result of purifying selection [23]. Renzette et al. suggested more generally that the relatively small genome sizes characterizing most viruses may shift the distribution of mutational fitness effects such that a greater proportion of new mutations are deleterious and therefore subject to purifying selection, as compared to humans for example. This, combined with the generally high virus mutation rates, results in widespread background selection effects [26].

## 5. Clinical Implications

High levels of diversity and multiple-strain infection have been found to be associated with higher viral loads [30,50,51], delayed clearance [52], coinfection with other herpesviruses [15], and faster disease progression in HCMV disease [15] and AIDS patients [53], as well as lethal outcomes during gestation [54]. Thus, characterizing the forces shaping the diversity of HCMV populations discussed above is a critical step in the development of therapies and effective vaccines. Continued research into the magnitude and mechanisms of population-level variation should elucidate the connections between strain diversity and increased pathogenicity, and the identification of sites or epitopes that are conserved across all compartments will aid in novel vaccine development.

Hage et al. recently demonstrated the ability to detect the presence of mutations conferring resistance to antiviral drugs in clinically sampled HCMV populations, suggesting the possibility of tracking the presence and frequency of a suite of known resistance mutations during the course of treatment during HCMV infection [24]. They also found compartmentalization between blood and the vitreal humour on the level of resistance mutations in a patient suffering from HCMV retinitis, indicating that compartments unaffected by antiviral drugs may provide reservoirs of viral diversity during periods of treatment. Combined with persistent gene flow or periodic admixture between compartments [22], this would permit re-settlement of drug-resistant or naïve HCMV variants within targeted compartments during and after treatment. 

Given that no current methods exist to prevent maternal-fetal transmission or reduce the severity of fetal infection [55], acting to modulate the population level processes underlying HCMV transmission may be the key to improved treatment. For example, clinically imposing stronger and more severe population bottlenecks during pregnancy (i.e., during congenital infection) may reduce genomic HCMV variability in the fetus, thus limiting the pool of standing variation on which selection may act and thereby potentially improving treatment outcomes. Advances in immunotherapy have produced treatments capable of reducing the rate of maternal transmission of other viruses, such as heptatitis B and HIV, and may prove a promising route for either preventing transmission or imposing more severe bottlenecks in HCMV as well [56,57]

## 6. Conclusions and Future Directions

HCMV generally persists in highly diverse populations, with diversity ranging by up to two orders of magnitude across the genome [26]. Complex demographic histories featuring multiple population bottlenecks, rapid population growth, gene flow between compartments, and admixture events during reinfection—as well as variable effects of positive and negative selection and variable rates of mutation and recombination across the genome—all contribute to variable diversity within and between hosts, compartments, and time points. Via high-throughput genomic sequencing of HCMV populations, much has been recently elucidated regarding these processes of HCMV infection, dissemination and evolution, but much yet remains to be studied. In particular, new data pairing HCMV samples from congenitally infected neonates with samples from the viral populations of their mothers across multiple compartments would shed light on the ways in which diversity is passed from mother to child during infection, and the degree to which observed variation in patients is a result of new mutations versus the transmission of pre-existing diversity owing to multiple virion infections or subsequent admixture. 

Given what we now know regarding the frequency of mixed infection, reinfection, and gene flow between compartments, the development of successful vaccines and treatments for HCMV may be very difficult and require a knowledge and incorporation of variants not just from the targeted compartment, but from all compartments contributing to within-patient diversity. Furthermore, Pokalyuk et al. demonstrated that genetic sampling at individual loci can result in the misclassification of mixed infections as single infections, and Garrigue et al. have also recently shown that even when looking at individual loci, traditional Sanger sequencing can miss even relatively high-frequency resistance mutations, highlighting the utility of high-throughput, genome-level characterization of HCMV infection. This is of particular importance given the emerging links between genetic diversity and patient outcome [28,58]. Yet, given the rapidly declining costs of high-throughput sequencing methods, genome-wide multi-compartment patient sequencing paired with newly developed virus-specific population genetic analyses may soon be accessible and affordable as part of routine medical care, and there is ample reason to be optimistic that improved treatment strategies may be on the near horizon. 

## Figures and Tables

**Table 1:** Summary of terms and definitions.

Term	Definition
Population bottleneck	A sharp reduction in the size of a population, followed by recovery
Gene flow	The transfer of genetic variation between structured populations
Genetic hitchhiking	Changes in allele frequency at sites linked to positively selected loci
Background selection	Reduction of genetic diversity at sites linked to negatively selected loci
